# The relation of location-specific epicardial adipose tissue thickness and obstructive coronary artery disease: systemic review and meta-analysis of observational studies

**DOI:** 10.1186/1471-2261-14-62

**Published:** 2014-05-04

**Authors:** Fu-Zong Wu, Kang-Ju Chou, Yi-Luan Huang, Ming-Ting Wu

**Affiliations:** 1 Department of Radiology, Section of Thoracic and Circulation Imaging, Kaohsiung Veterans General Hospital, Kaohsiung, Taiwan; 2 Centre for Evidence-Based Medicine, Kaohsiung Veterans General Hospital, Kaohsiung, Taiwan; 3 Faculty of Medicine, School of Medicine, National Yang Ming University, Taipei, Taiwan; 4 Department of Internal Medicine, Kaohsiung Veterans General Hospital, Kaohsiung, Taiwan

**Keywords:** Coronary artery disease, Location-specific epicardial adipose tissue thickness, Meta-analysis

## Abstract

**Background:**

There is growing evidence about the importance of epicardial adiposity on cardiometabolic risk. However, the relation of location-specific epicardial adipose tissue (EAT) thickness to coronary atherosclerotic burden is still unclear.

**Methods:**

This meta-analysis was used to study the relations between location-specific EAT thickness and obstructive coronary artery disease (CAD). A systemic literature search to identify eligible studies that met the inclusion criteria from the beginning until January 2014 was made. We conducted the meta-analysis of all included 10 published studies. Pre-specified subgroup analyses were performed according to ethnicity, body mass index, diagnostic tools for CAD, and measurement tool if presence of high heterogeneity between studies. Potential publication bias was also assessed.

**Results:**

We identified ten observed studies with a total of 1625 subjects for planned comparison. With regard to the association between obstructive CAD and location-specific EAT thickness at the right ventricular free wall, caution is warranted. The pooled estimate showed that location-specific EAT thickness at the right ventricular free wall was significantly higher in the CAD group than non-CAD group (standardized mean difference (SMD): 0.70 mm, 95% CI: 0.26-1.13, P = 0.002), although heterogeneity was high (I^2^ = 93%). It should be clear that only the result of echocardiography-based studies showed a significant association (SMD: 0.98 mm, 95% CI: 0.43-1.53, P = 0.0005), and the result of all included CT-based studies showed a non-significant association (SMD: 0.06 mm, 95% CI: -0.12-0.25, P = 0.50). In the subgroup analysis, the “diagnostic tools for CAD” or “measurement tool of EAT thickness” are potential major sources of heterogeneity between studies. With regard to location-specific EAT thickness at the left atrioventricular (AV) groove, it was significantly higher in the CAD group than non-CAD group (SMD: 0.74 mm, 95% CI: 0.55-0.92, P <0.00001; I^2^ = 0%).

**Conclusion:**

Our meta-analysis suggests that significantly elevated location-specific EAT thickness at the left AV groove is associated with obstructive CAD. Based on the current evidence, the location-specific EAT thickness at the left AV groove appears to be a good predictor in obstructive CAD, especially in Asian populations. Furthermore well-designed studies are warranted because of the current limited number of studies.

## Background

Excessive epicardial adipose tissue (EAT) accumulation within the pericardial sac has been suggested to play an important role in the development of coronary artery atherosclerosis through potential paracrine or endocrine mechanism by exerting inflammatory mediators such as TNF-alpha, IL-6, adipocytokines, and leptin [1-5]. Several previous studies have found that CT-measured EAT volume played an important causal role in coronary atherosclerotic burden or coronary artery disease [6-8].

There is increasing attention on the location-specific EAT thickness as a potential predictor in cardiometabolic disease because of uneven regional distribution of EAT around the heart, especially mostly located in the atrioventricular (AV) groove and interventricular (IV) groove [9-11]. Echocardiographic EAT measurement is a non-invasive and simple way to measure location-specific EAT at the right ventricular free wall clinically [12-17]. Alternatively, CT (computed tomography) or MRI (magnetic resonance imaging) can also provide the simple but precise 2D measurement in the thickness of EAT in all cardiac segments [11,18,19]. Current evidence about the association between location-specific EAT thickness at the right ventricular free wall and coronary artery disease (CAD) is still controversial. Several studies using echocardiography have demonstrated a significant relationship between CAD and location-specific EAT thickness measured at right ventricular free wall [13,14,16,17], while some studies using echocardiography or CT failed to observe a significant association [11,12,19,20]. Recent evidence has suggested that there is increasing attention on location-specific EAT thickness at the left AV groove as a potentially new biomarker associated with cardiometabolic risks by using 2D CT or MR measurement [11,19,21,22]. In this study, we conducted a meta-analysis to study the relationship between location-specific EAT thickness and obstructive CAD, which may serve as a reliable predictor and improve CAD risk stratification in the future.

## Methods

This meta-analysis was conducted according to the Meta-analysis Of Observational Studies in Epidemiology (MOOSE) statement [23].

### Search strategy

We searched the Pubmed, Ovid Medline, Ovid Embase and Cochrane databases for relevant articles. Reviews were independently performed by two authors using the following search terms: “epicardial adipose tissue”, “epicardial fat”, “subepicardial adipose tissue”, “subepicardial fat”, “coronary artery disease [Mesh]”, “atherosclerosis [Mesh]”, and “cardiovascular disease [Mesh]”, from the beginning of publication until January 2014. No language restrictions were applied. The pre-specified inclusion criteria were as follows: (a) observational studies to investigate the relationship between location-specific EAT thickness and CAD measured by echocardiography, CT or MRI-based quantitative measurements; (b) subjects with suspicion of CAD or at high risk of CAD clinically, and then subsequently divided into CAD and non-CAD groups assessed by coronary angiography, coronary CT angiography and coronary MR angiography. CAD group was defined as luminal stenosis ≥ 50% in one or more coronary arteries; and (c) for each study, the mean difference, standard deviation (SD) and sample size of subjects of CAD and non-CAD groups were reported in the literature. References within studies that met the inclusion criteria were reviewed for any possible missing relevant articles. Studies were excluded if any of the inclusion criteria were not met. All 10 published studies that met these inclusion criteria were considered eligible for further meta-analysis.

### Data extraction and quality assessment

Data were abstracted and quality of studies was assessed independently by two reviewers (F.Z.W. and M.T.W.). Disagreement on specific studies between the two reviewers was resolved by consensus. Data were extracted from each study including the sample size and mean difference ± SD in location-specific EAT thickness in both CAD and non-CAD groups (regrouping and calculating the mean difference and mixed SD in the study of Eroglu et al. [14]). Methodological study quality was assessed using the Strengthening of Reporting of Observational Studies in Epidemiology (STROBE) checklist of 22 items [24].

### Data analysis

All statistical analyses were performed using the Comprehensive Meta-Analysis version 2 (Biostat, Englewood, New Jersey) and the Review Manager 5.0 software, available through the Cochrane Collaboration. Both fixed- and random-effects models were used for analysis of the standardized mean difference (SMD) in the CAD and non-CAD group. Heterogeneity was assessed and quantified using Cochran’s Q statistic and the I^2^ statistic [25]. If I^2^ was below 30%, fixed effects were chosen; if I^2^ was equal to or greater than 30%, then random effects were applied [26]. Before the analysis, we formed a prior hypothesis that if heterogeneity existed in the studies, the source could be due to: (1) diagnostic tool for CAD (coronary angiography vs. coronary CT angiography); (2) ethnicity (Asian vs. non-Asian); (3) measurement tool of EAT (echocardiography vs. CT); and (4) body mass index (BMI). We also performed subgroup and univariate meta-regression analyses to investigate potential sources of variability. Publication bias was assessed if available by using Egger’s test to examine the likely presence of publication bias.

## Results

### Study identification and selection

Overall, 312 articles were initially identified according to the broad criteria. After the initial screening of the title and abstract, 21 potentially relevant articles were retrieved for detailed review of their full text. Many studies that investigated the association between epicardial fat volume and CAD were excluded in this step.

**Figure 1:**
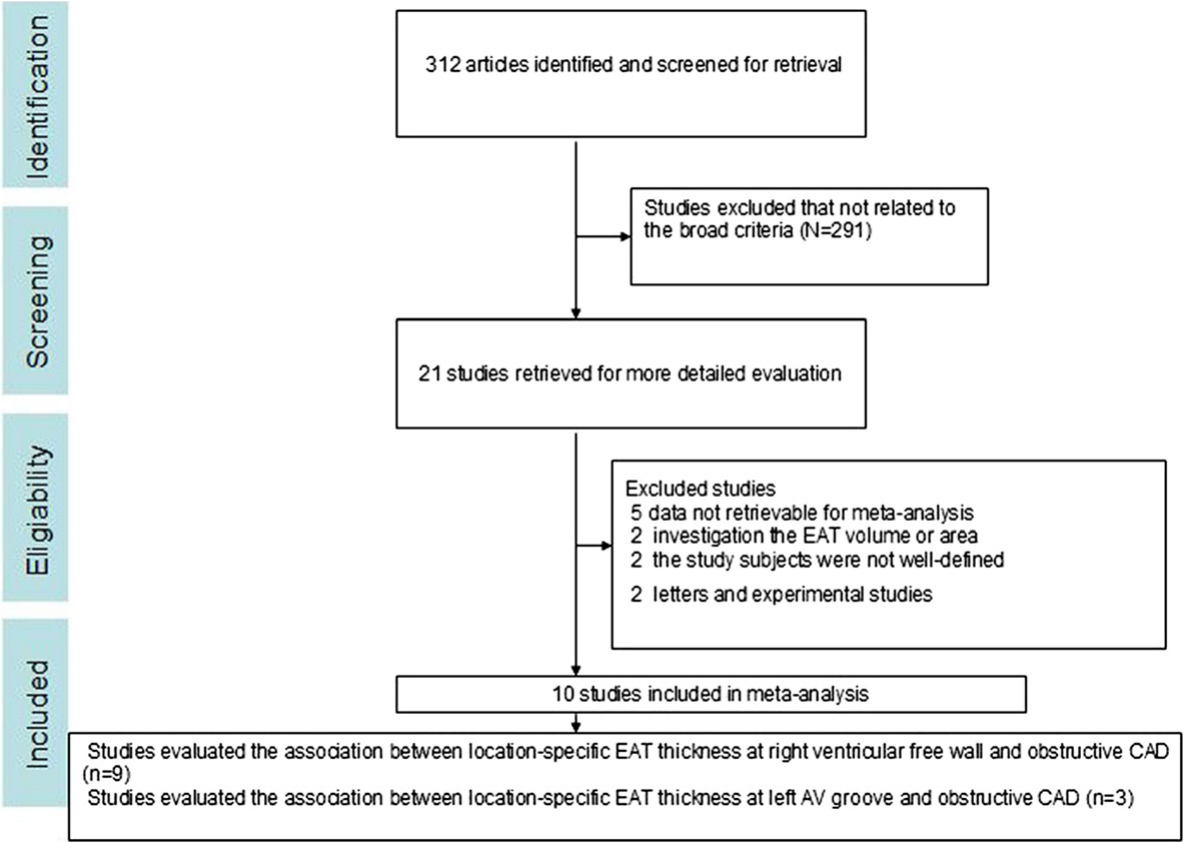
**Flowchart of study selection process to determine the studies to be included in the meta-analysis.** EAT, epicardial adipose tissue.

In all these relevant studies, 11 studies were excluded after the detail review of their full text. Of these 11 studies, five studies were excluded because they did not report the detailed data for the meta-analysis [27-31]; two studies were excluded because of an investigation of the relationship between the EAT volume or area with CAD [32,33]; two studies were excluded because the study subjects were not well-defined or did not meet the criteria [15,34]; and the remaining two were letters and experimental studies [5,35]. The remaining 10 observational studies were eligible for the meta-analysis [11-14,16,17,19-21,36]. The flowchart of the search and selection of studies in this meta-analysis is illustrated in Figure 1. Overall, these studies included 1625 patients (924 subjects with CAD, and 701 subjects without CAD).

Table 1 summarize the characteristics of the nine observational studies investigating the association between location-specific EAT thickness at the right ventricular free wall and obstructive CAD. Table 2 summarizes the characteristics of the three observational studies investigating the association between location-specific EAT thickness at the left AV groove and obstructive CAD.

**Table 1:** Demographics of included studies in the association between location-specific EAT thickness at the right ventricular free wall and obstructive CAD

			**CAD group**			**Non-CAD group**							
			**EAT thickness (mm)**			**EAT thickness (mm)**							
**References**	**Time**	**No. of patients**	**Mean**	**SD**	**Total**	**Mean**	**SD**	**Total**	**BMI (kg/m**^**2**^**)**	**Diagnostic tool**	**Measurement tool**	**Ethnicity**	**Study quality**
Chaowalit et al. [12]	2006	139	2.22	1.86	85	2.16	1.77	54	28.7 ± 5.5	Coronary angiography	Echocardiography	Non-Asian	20
Yun et al. [13]	2009	153	3.8	1.9	85	1.8	1.4	68	25.2 ± 3.1	Coronary angiography	Echocardiography	Asian	21
Eroglu et al. [14]	2009	150	7.3	1.2	80	4.68	1.13	70	28.9 ± 4.5	Coronary angiography	Echocardiography	Non-Asian	21
Wang et al. [11]	2010	224	4.3	1.8	140	4.1	2.0	84	26.1 ± 3.3	Coronary angiography	CT	Asian	21
Bastarrika et al. [20]	2010	45	8.57	2.08	23	7.69	2.68	22	29.1 ± 5.7	Coronary angiography	CT	Non-Asian	20
Mustelier et al. [16]	2011	250	6.6	2.8	185	4.7	2.3	65	27.4 ± 5.3	Coronary angiography	Echocardiography	Non-Asian	19
Shemirani et al. [17]	2012	292	5.4	1.9	171	4.4	1.8	121	NR	Coronary angiography	Echocardiography	Non-Asian	19
Wu et al. [19]	2013	208	5.8	2.3	97	5.9	2.3	111	24.8 ± 3.3	Coronary CT angiography	CT	Asian	21
Kaya et al. [36]	2013	64	6.43	0.9	34	5.35	0.75	30	26.0 ± 3.0	Coronary angiography	Echocardiography	Non-Asian	19

BMI, body mass index; EAT, epicardial adipose tissue; CAD, coronary artery disease; CT, computed tomography; NR, not reported.

**Table 2:** Demographics of included studies in the association between location-specific EAT thickness at left AV groove and obstructive CAD

			**CAD group**			**Non-CAD group**							
			**EAT thickness (mm)**			**EAT thickness (mm)**							
**References**	**Time**	**No. of patients**	**Mean**	**SD**	**Total**	**Mean**	**SD**	**Total**	**BMI (kg/m**^**2**^**)**	**Diagnostic tool**	**Measurement tool**	**Ethnicity**	**Study quality**
Wang et al. [11]	2010	224	13.2	3.1	140	11.3	2.5	84	26.1 ± 3.3	Coronary angiography	CT	Asian	21
Kim et al. [21]	2012	100	13	2.6	24	11.5	2.1	76	25.3 ± 3.2	Coronary MR angiography	MRI	Asian	21
Wu et al. [19]	2013	208	17.8	3.9	97	14.3	4.3	111	24.8 ± 3.3	Coronary CT angiography	CT	Asian	21

BMI, body mass index; EAT, epicardial adipose tissue; CAD, coronary artery disease; CT, computed tomography; AV, atrioventricular.

### Location-specific EAT thickness at the right ventricular free wall and obstructive CAD

With regard to the association between the location-specific EAT thickness at the right ventricular free wall and obstructive CAD, there was a significant difference in the SMD between CAD and no-CAD groups (0.70 mm, 95% CI: 0.26-1.13, p = 0.002) in a random-effect pooled analysis of these studies (fixed-effect model in the Additional file 1: Figure S1). Statistically significant heterogeneity was observed in the study results (p < 0.00001, I^2^ = 93%). There was no indication of publication bias with Egger’s test (p = 0.229, funnel plot shown in Additional file 2: Figure S2). A sensitivity analysis, in which one study was removed at a time, was performed to evaluate the stability of the results. Results were similar in all sensitivity analyses.

**Figure 2:**
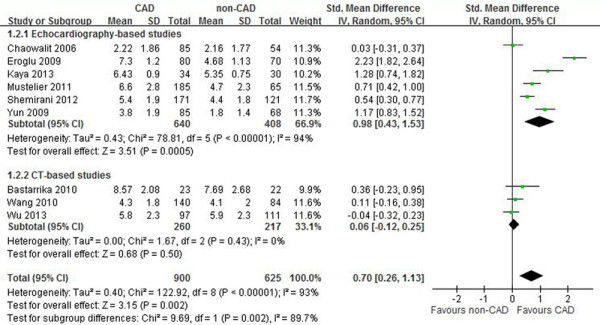
**Forest plot for SMD (random-effect model) in location-specific EAT thickness at the right ventricular free wall between CAD and non-CAD group in the overall meta-analysis (including nine published studies).** In addition, subgroup analyses were assessed by the measurement tool of EAT thickness (echocardiography or CT). SMD, standardized mean difference; CAD, coronary artery disease; EAT, epicardial adipose tissue.

**Figure 3:**
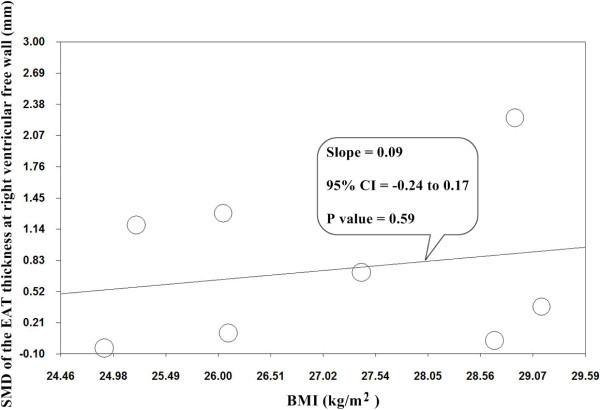
**Meta-regression analysis between BMI and SMD of location-specific EAT thickness at right ventricular free wall.** BMI, body mass index; SMD, standardized mean difference; EAT, epicardial adipose tissue.

Subgroup analyses were performed in an attempt to explore the possible causes of heterogeneity, according to ethnicity, diagnostic tools for CAD, and measurement tool of EAT thickness. In subgroup analysis according to ethnicity shown in Table 3, the test for subgroup differences did not show a significant interaction. In contrast, the test for subgroup differences showed a significant interaction in the subgroup analysis according to diagnostic tools for CAD. In addition, the test for subgroup differences showed a significant interaction according to measurement tool of EAT thickness. In the subgroup of echocardiography-based studies, the random-effects model was used for the analysis, because the test for heterogeneity was statistically significant (p < 0.00001; I^2^ = 94%). There was a significant difference in the SMD between CAD and no-CAD groups (0.98 mm, 95% CI: 0.43-1.53, p = 0.0005). By contrast, there was no significant difference in the SMD between CAD and no-CAD groups (0.06 mm, 95% CI: -0.12-0.25, p = 0.50) in the subgroup of CT-based studies, and greater homogeneity was also observed within this subgroup shown in Figure 2 (p = 0.43; I^2^ = 0). Therefore, diagnostic tools for CAD and measurement tool of EAT thickness were the potential sources of heterogeneity between studies. In univariate random effects meta-regression analysis, BMI was not significantly associated with effect size shown in Figure 3 (95% CI: -0.24 -0.17, p = 0.59).

**Table 3:** Subgroups analyses according to the ethnicity, diagnostic tool, and measurement tool comparing location-specific EAT thickness at the right ventricular free wall in subjects of CAD and non-CAD groups

**Subgroups**	**Studies**	**Subjects**	**SMD (95% CI)**	**p Value**
Ethnicity				
Asian	3	585	0.4 (-0.28 to 1.09)	0.36
Non-Asian	6	940	0.80 (0.30 to 1.29)	
Diagnostic tool				
Coronary angiography	8	1317	0.75 (0.34 to 1.16)	0.002
Coronary CT angiography	1	208	-0.04 (-0.23 to 0.32)	
Measurement tool				
Echocardiography	6	1048	0.98 (0.43 to 1.53)	0.002
CT	3	477	0.06 (0.17 to 1.09)	

p Value: test of interaction between subgroups. CAD, coronary artery disease; SMD, standardized mean difference; CT, computed tomography.

### Location-specific EAT thickness at the left AV groove and obstructive CAD

**Figure 4:**
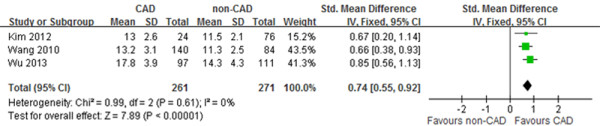
**Forest plot for SMD in location-specific EAT thickness at the left AV groove between CAD and non-CAD groups in the meta-analysis (including three published studies).** SMD, standardized mean difference; CAD, coronary artery disease; EAT, epicardial adipose tissue; AV, atrioventricular.

Figure 4 shows the forest plot of the pooled results of the SMD of the three studies in order to investigation the association between obstructive CAD and EAT thickness at the left AV groove. In a fixed-effect pooled analysis, there was a significant difference in the SMD between CAD and no-CAD groups (0.74 mm, 95% CI: 0.55-0.92, p < 0.00001), and low heterogeneity among the three studies shown in Figure 4 (p = 0.61, I^2^ = 0%). Overall, location-specific EAT thickness at the left AV groove was significantly thicker in the CAD group than the no-CAD group. Publication bias was not assessed because of limited studies.

## Discussion

### The role of location-specific EAT thickness at the right ventricular free wall in obstructive CAD

With regard to the association between the location-specific EAT thickness at the right ventricular free wall and obstructive CAD, the overall results of the meta-analysis in nine studies show a statistically significantly increase in EAT thickness at the right ventricular free wall in the CAD group than in the no-CAD group.

It should be clear that only the result of echocardiography-based studies (six studies) showed a significant association between CAD and the EAT thickness at right ventricular free wall, and the result of all included CT-based studies (three studies) showed a non-significant association. And low between-study heterogeneity was also observed in the CT-based studies (three studies) assessed at the right ventricular free wall. However, extreme between-study heterogeneity was also observed in the overall meta-analysis (nine studies) and echocardiography-based studies (six studies). Therefore, all this evidence suggested that CT-based measurement may potentially be a more reliable tool than echocardiography-based measurement.

Regarding the association between location-specific EAT thickness at the right ventricular free wall and obstructive CAD, subgroup and meta-regression analyses for all covariates have been used to address the issues of heterogeneity between studies. A recent meta-analysis of worldwide study demonstrates a significant correlation between EAT and BMI, but shows a relationship between EAT and metabolic syndrome independent of BMI [37]. These findings support the meta-regression analysis in our study that revealed BMI was not considered a potential source of heterogeneity between eight studies.

We think a possible explanation for the high heterogeneity between studies was that the difference in reliability and reproducibility between the two tools (echocardiography vs. CT) should be seriously concerned. In comparison with CT-based measurement, echocardiography is a non-invasive imaging modality in EAT measurement that requires less cost and is less time-consuming. However, a recent study has shown the poor reproducibility of echocardiographic EAT measurements assessed by intraclass correlation coefficient, and low concordance observed between CT and echocardiographic measurement [38]. One the contrary, several studies have demonstrated high interobserver reliability and intraobserver reproducibility of EAT thickness measured by CT [11,39,40]. Potential measurement bias in the EAT measurement by echocardiography may contribute to the disparate results between echocardiography-based and CT-based studies. And recent longitudinal studies have also demonstrated non-association between EAT thickness assessed by echocardiography and cardiac events or major adverse cardiac events (MACE) [29,31], which also supports the negative result by CT-based studies in the subgroup analysis. Therefore, we suggest that the effect of location-specific EAT at the right ventricular free wall on CAD is still uncertain. However, future well-designed studies are warranted because of limited CT-based studies measuring location-specific EAT thickness at the right ventricular free wall.

### The role of location-specific EAT thickness at the left AV groove in obstructive CAD

With regard to the association between the location-specific EAT thickness at the left AV groove and obstructive CAD, the results from the current meta-analysis indicate increased location-specific EAT thickness at the left AV groove was associated with obstructive CAD assessed by CT or MRI.

And high homogeneity between studies was observed, despite diagnosis of CAD in different imaging modalities, including coronary angiography, coronary CT angiography and MR coronary angiography. Substantial evidence has also supported the diagnostic accuracy of coronary CT angiography and MR coronary angiography in the evaluation of coronary arteries stenosis [41-43].

While there is no obvious explanation for this observation, uneven regional distribution of EAT around the heart may provide clues. Recent studies also suggest that location-specific EAT thickness at the left AV groove may play a important causal role in cardiometabolic disease through a possible mechanism described by Wang et al. [11] whereby diffusion of EAT paracrine metabolites through thin-walled coronary venous networks into the coronary sinus is due to abundant coronary venous sinus drainage networks in the left AV groove. In addition, location-specific EAT thickness at the left AV groove can be easily and reliably assessed in 2D-based CT or MRI images available in routine clinical practice for cardiovascular risk assessment [11,19,21]. Taken together, these findings may indicate that combined measurement of EAT thickness in the left AV groove and coronary calcium score provides more in-depth information than conventional risk factor assessment in CAD prediction [44]. Because of the limited number of studies almost exclusively in Asian populations, future well-designed cohort studies are warranted to investigate the cause-effect relationship by CT or MRI.

### Study strengths and limitations

It is our belief that the present study has demonstrated certain advantages over the previous meta-analysis [45]. First, in contrast to previous meta-analysis by Xu et al. [45] on this topic, we used explicit criteria to define inclusion criteria. To investigate the association between regional EAT thickness and CAD, we select the location-specific EAT thickness at the right ventricular free wall or left AV groove by echocardiography, CT or MRI rigorously. However, the previous meta-analysis has used these data of the “average” EAT thickness extracted from the study by Wang et al. [11]. Second, the aims of the previous meta-analysis and our study were both to investigate the relationship between EAT thickness and CAD. We found one included study in the previous meta-analysis that investigated the relationship between regional EAT thickness and descending thoracic aorta atherosclerosis [46], instead of all included nine studies rigorously assessing obstructive CAD in our study. Third, study quality was assessed using the STROBE statement to ensure that all studies reach a level of quality in this meta-analyses.

The current study had several potential limitations. First, all ten included studies in the present meta-analysis were case-control or cross-sectional studies despite assessing study quality using the STROBE statement, which has limitations in providing definite information about the cause-and-effect relationship. In addition, the best evidence comes primarily from meta-analyses of randomized controlled trials. Second, there are many confounding factors associated with CAD, such as the Framingham risk score and CAC score were not considered in this meta-analyses. However, we have investigated a number of factors to explore between-study heterogeneity. The included studies were different in diagnostic tools for CAD and measurement tools of EAT, which may contribute to the heterogeneity among the studies. Third, there are limited studies in assessing the association between left AVG EAT thickness and obstructive CAD despite recognition that homogeneity exists between studies, which may lead to insufficient evidence to support the result.

## Conclusion

In summary, our meta-analysis suggests that significantly elevated location-specific EAT thickness at the left AV groove is associated with obstructive CAD, and homogeneity influenced agreement between studies. Based on the current evidence, the location-specific EAT thickness at the left AV groove appears to play an influential role in the prediction of obstructive CAD, especially in Asian populations. In addition, adding EAT thickness at the left AV groove on top of clinical CAD risk factors plus Agatston score may provide further evidence in predicting CAD in the population with suspicion of CAD [19,44]. Subsequent, large, more definitive studies are needed to confirm these findings and to establish if location-specific EAT thickness at the left AV groove be used in clinical context.

## Abbreviations

AV: Atrioventricular; BMI: Body mass index; CAC: Coronary arterial calcification; CAD: Coronary artery disease; CT: Computed tomography; EAT: Epicardial adipose tissue; MRI: Magnetic resonance imaging; MOOSE statement: Meta-analysis Of Observational Studies in Epidemiology statement; SD: Standard deviation; SMD: Standardized mean difference; STROBE checklist: Strengthening of Reporting of Observational Studies in Epidemiology checklist; MACE: Major adverse cardiac events.

## Competing interests

The authors declare that they have no competing interests.

## Authors’ contributions

FZW and MTW participated in the conception, design of the study, collected the data, performed statistical analyses, and drafted the manuscript. YLH and KJC helped to collect data and draft the manuscript. All authors read and approved the final manuscript.

## Pre-publication history

The pre-publication history for this paper can be accessed here:

http://www.biomedcentral.com/1471-2261/14/62/prepub

## Supplementary Material

Additional file 1: Figure S1 Forest plot for SMD (fixed-effect model) in location-specific EAT thickness at the right ventricular free wall between CAD and non-CAD groups in the overall meta-analysis (including nine published studies). Moreover, subgroup analyses were assessed by the measurement tool of EAT thickness (echocardiography or CT). SMD, standardized mean difference; CAD, coronary artery disease; EAT, epicardial adipose tissue. Click here for file

Additional file 2: Figure S2 Funnel plot for potential publication bias in the overall meta-analysis of the association between location-specific EAT at the right ventricular free wall and obstructive CAD, including nine studies. EAT, epicardial adipose tissue; CAD, coronary artery disease. Click here for file
