# Supplementary figures and images for: The relation of location-specific epicardial adipose tissue thickness and obstructive coronary artery disease: systemic review and meta-analysis of observational studies
Source: BMC Cardiovasc Disord. 2014 May 4;14:62. doi: 10.1186/1471-2261-14-62 (PMC4101835; doi:10.1186/1471-2261-14-62)

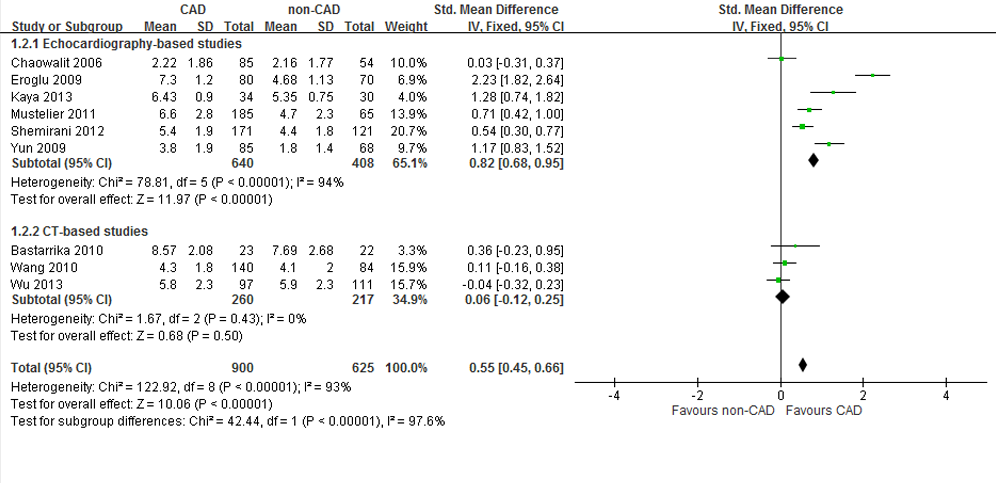

Supplement: Additional file 1: Figure S1 — Forest plot for SMD (fixed-effect model) in location-specific EAT thickness at the right ventricular free wall between CAD and non-CAD groups in the overall meta-analysis (including nine published studies). Moreover, subgroup analyses were assessed by the measurement tool of EAT thickness (echocardiography or CT). SMD, standardized mean difference; CAD, coronary artery disease; EAT, epicardial adipose tissue. [file 1471-2261-14-62-S1.tiff]

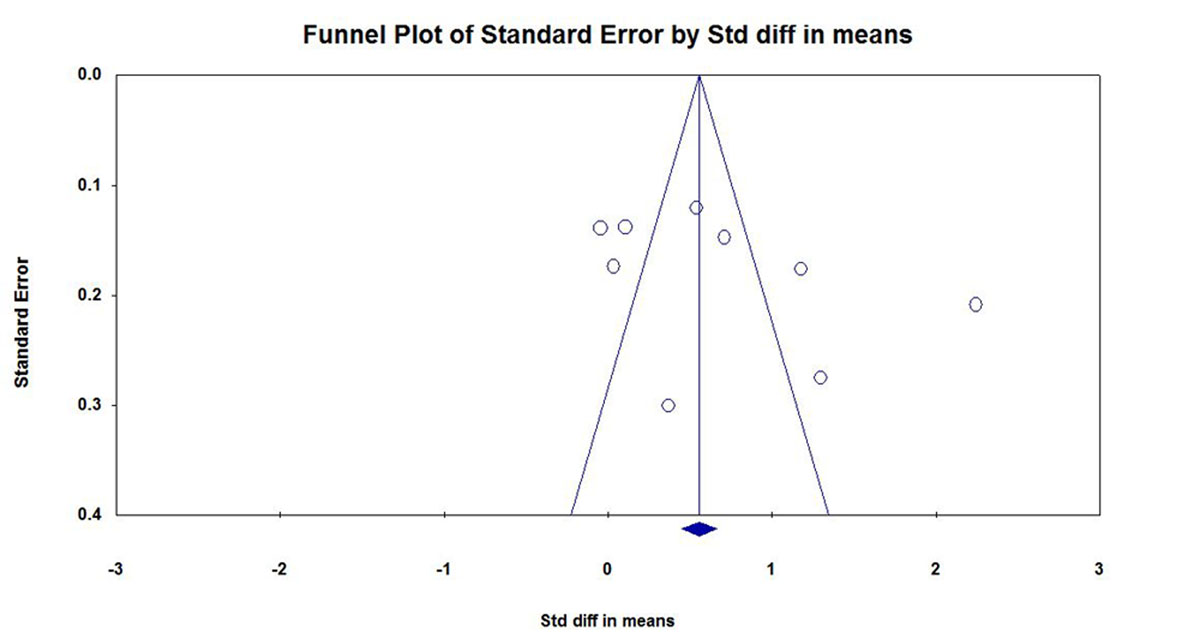

Supplement: Additional file 2: Figure S2 — Funnel plot for potential publication bias in the overall meta-analysis of the association between location-specific EAT at the right ventricular free wall and obstructive CAD, including nine studies. EAT, epicardial adipose tissue; CAD, coronary artery disease. [file 1471-2261-14-62-S2.jpeg]
